# Development and validation of a (semi-)quantitative UHPLC-MS/MS method for the determination of 191 mycotoxins and other fungal metabolites in almonds, hazelnuts, peanuts and pistachios

**DOI:** 10.1007/s00216-013-6831-3

**Published:** 2013-03-08

**Authors:** Elisabeth Varga, Thomas Glauner, Franz Berthiller, Rudolf Krska, Rainer Schuhmacher, Michael Sulyok

**Affiliations:** 1 Christian Doppler Laboratory for Mycotoxin Metabolism and Center for Analytical Chemistry, Department for Agrobiotechnology (IFA-Tulln), University of Natural Resources and Life Sciences, Vienna (BOKU), Konrad Lorenz Str. 20, 3430 Tulln, Austria; 2 Chemical Analysis Group, Agilent Technologies Sales & Services GmbH & Co. KG, Hewlett-Packard-Str. 8, 76337 Waldbronn, Germany

**Keywords:** Multi-target analysis, Tandem mass spectrometry, Ultra-high-performance liquid chromatography, Nuts

## Abstract

A multi-target method for the determination of 191 fungal metabolites in almonds, hazelnuts, peanuts and pistachios was developed. The method includes all mycotoxins regulated in the European Union and mycotoxins regularly found in food. After extraction with an acidified acetonitrile water mixture, the raw extract was diluted and injected directly into the UHPLC-MS/MS system. In two chromatographic runs, analysis was performed in positive and in negative ionisation mode. The method was in-house validated for the most important 65 analytes in these four commodities. Apparent recoveries between 80 and 120 % were obtained for about half of the analyte–matrix combinations. Good repeatabilities (standard deviations < 10 %) were achieved for the vast majority (83 %) of all cases. Only in 6 % of all combinations did the standard deviations exceed 15 %. Matrix effects, arising during electrospray ionisation, significantly influenced the determination. For instance, signal suppression was observed for several early-eluting analytes and also signal enhancement up to 295 % for physcion in peanuts was determined. Concerning extraction recovery, 94 % of the analyte–matrix combinations showed values higher than 50 %. Lower limits of quantification ranged between 0.04 μg kg^−1^ for enniatin B3 in peanuts and 500 μg kg^−1^ for HC toxin in hazelnuts. Additionally, the applicability of the developed method was demonstrated through the analysis of 53 naturally contaminated nut samples from Austria and Turkey. Overall, 40 toxins were quantified; the most frequently found mycotoxins were beauvericin (79 %), enniatin B (62 %) and macrosporin (57 %). In the most contaminated hazelnut sample, 26 different fungal metabolites were detected.

## Introduction

Mycotoxins are secondary fungal metabolites which are found in a broad range of food and feed, such as cereals, spices, coffee, nuts or dried fruits [1]. They have the capability of causing acute toxic, carcinogenic, mutagenic, teratogenic, immunotoxic or oestrogenic effects in animals and humans [2]. Mycotoxins show a huge structural diversity resulting in a variety of chemical and physicochemical properties. The most important mycotoxins in food and feed, which are regulated in the European Union [3], are aflatoxins (AFB_1_, AFB_2_, AFG_1_, AFG_2_); ochratoxin A (OTA); type A and B trichothecenes (e.g. HT-2 toxin, T-2 toxin and deoxynivalenol (DON)); fumonisins; and zearalenone.

During the last years, single analyte methods for the detection and quantification of mycotoxins are more and more replaced by multi-target methods for the simultaneous determination of different yet co-occurring classes of mycotoxins. The majority of these methods are based on the combination of high- or ultra-high-performance liquid chromatography with tandem (e.g. [4–7]) or high-resolution [8] mass spectrometry. The importance of mass spectrometry for the confirmation of mycotoxin identity is emphasised, e.g. in [1]. A major advantage of LC-MS-based multi-target methods is the increased sample throughput compared to single-analyte methods. Still, such multi-target methods have to cope with huge differences in the relevant toxin concentrations. Naturally, the choice of an appropriate extraction solvent for a large number of analytes with different physicochemical properties is also a great challenge. Mixtures of water with high amounts of methanol or acetonitrile (>75 %) are appropriate extraction solvents for most mycotoxins. However, for fumonisins, higher extraction recoveries are achieved when the water proportion is increased and/or the pH of the solvent is decreased [4]. Mol et al. [9] concluded that aqueous acetone is the favourite extraction solvent with respect to extraction recovery, but aqueous acetonitrile should be preferred considering matrix effects. Extractions using aqueous methanol resulted in extracts exhibiting both lower extraction recoveries and causing more severe matrix effects [9]. Anyway, the chosen solvent has to be a compromise to reach agreeable extraction recoveries for the majority of the analytes.

Regarding cleanup, multi-target methods use simple “dilute-and-shoot” approaches [4, 5, 7, 10], solid phase extraction [6, 11, 12], MycoSep® columns [13], immunoaffinity columns [14] and, recently, also QuEChERS [8, 15, 16]. A drawback of most multi-target methods is that they require extensive validation which is time- and cost-consuming and, hence, often reduced to a minimum. Therefore, these methods are often used just for semiquantitative screening purposes. Most multi-target methods were developed for the measurement of raw cereals, whereas data on the performance characteristics in other matrices like nuts are scarce.

Nuts are hard-shelled fruits of some plants having an indehiscent seed and are an important source of nutrients for humans and animals. Almonds, hazelnuts and pistachios belong to the most popular tree nuts [17]. More correctly, almonds are not nuts, but drupes which consist of a hull and a hard shell containing the seed. Peanuts or groundnuts are actually legumes, but in the general linguistic usage, they are referred to as nuts. For simplification, the term “nut” will be used in this paper for all four matrices under investigation (almonds, hazelnuts, peanuts and pistachios).

Fungi of the genera *Fusarium*, *Alternaria* and *Cladosporium* dominate the mycobiota of nuts in the field, whilst in storage, *Aspergillus*, *Penicillium* and *Trichoderma* are predominant [18]. Almost all *Aspergillus parasiticus* strains tested in tree nuts produced aflatoxins, whilst all of the detected *Aspergillus alliaceus* strains produced OTA [19]. In general, nuts with thick shells (e.g. macadamia nuts) are better protected against the intrusion of moulds. Other nuts, like pistachios, are more prone to mould infestation due to shell splitting at the end of maturation [20]. Sorting and elimination of split nuts can decrease the contamination of mycotoxins in the lot significantly [21]. Information about mycotoxin contamination of nuts other than aflatoxins (recently reviewed in [22]) and OTA is limited. In most cases, only single (OTA) [23] or group-target (aflatoxins) [24] methods using chromatographic (TLC, LC-FLD or LC-MS/MS) or immuno-based methods (ELISA, fluorescence immunoassays) were applied for the investigation of the content of those mycotoxins. In 2008, Spanjer et al. [5] published the validation of an LC-MS/MS-based multi-target method for the determination of mycotoxins in various matrices, including peanuts (13 mycotoxins) and pistachios (24 mycotoxins). A first application for a semiquantitative screening of multiple mycotoxins in various food (including six nut samples) was published in 2010 [25]. Other studies investigated the occurrence of mycotoxins in peanut cake [26, 27] and peanuts [28] from Africa.

Based on previous HPLC-MS/MS methods for the multiple determination of mycotoxins in cereals developed in our group [4, 7], we created a new multi-target ultra-high-performance liquid chromatographic tandem mass spectrometric (UHPLC-MS/MS) method which covers a total number of 191 fungal metabolites. Validation parameters were obtained for those analytes which seem to be of importance in almonds, hazelnuts, peanuts and pistachios. Finally, we show the applicability of the method by analysing nuts bought on Austrian and Turkish markets.

## Experimental

### Chemicals, standards and samples

Methanol and acetonitrile (both LC gradient grade), as well as acetic acid (p.a.), were purchased from VWR International (Vienna, Austria), whereas ammonium acetate (MS grade) was obtained from Sigma-Aldrich (Vienna, Austria). Water was purified by reverse osmosis followed by a Milli-Q Plus system from Millipore (Molsheim, France).

Solid standards or stock solutions were collected from different sources over the years: They were either isolated by our own group, provided by other research groups or purchased from one of the following commercial companies: Alexis Austria (Vienna, Austria), Alfarma (Prague, Czech Republic), Axxora Europe (Lausanne, Switzerland), Bioaustralis (distributed by Tebu-Bio, Germany), Iris Biotech GmbH (Marktredwitz, Germany), LGC Promochem GmbH (Wesel, Germany), Romer Labs (Tulln, Austria) and Sigma-Aldrich. Individual stock solutions of the standards were prepared by dissolving the weighted solid substance in acetonitrile, or, if insoluble, either in methanol, acetonitrile/water (50:50, *v*/*v*), methanol/water (50:50, *v*/*v*) or pure water. The stock solutions were combined to 30 working solutions containing up to 13 individual mycotoxins and were stored at −20 °C. Before usage, the working solutions were brought to room temperature in the dark, thoroughly mixed and a multi-analyte stock solution was freshly prepared thereof. Neat standard solutions covering a concentration range of three orders of magnitude were obtained by dilution of the multi-analyte stock solution with dilution solvent (acetonitrile/water/acetic acid, 20:79:1, *v*/*v*/*v*) resulting in relative concentrations of 1:3.33:10:33.3:100:333:1,000.

Nut samples were purchased from various stores in Tulln and Vienna (both Austria) or were kindly provided by Dr. Ahmet D. Duman from the Department of Food Engineering of the Faculty of Agriculture of Kahramanmaras Sutcu Imam University (KSU), Turkey. Samples obtained in Austria were stored at −20 °C on the same or following day until usage. Turkish samples were collected on markets, orchards and in warehouses from the Black Sea Region (hazelnuts) or from the Osmaniye province (peanuts) in the harvest season of 2007. Turkish samples were frozen at −20 °C, sent cooled to the IFA-Tulln on the day of purchase and stored until measurement at −20 °C.

### Sample preparation

Sample preparation was based on the method for cereals described by Sulyok et al. [4]. In-shell pistachios were pealed and nut samples were ground using an Osterizer® Blender (Sunbeam Oster Household Products, USA). Of the ground samples, 5.00 ± 0.01 g was weighted in 50-mL polypropylene tubes (Sarstedt, Wr. Neudorf, Austria). For extraction, 20 mL extraction solvent (acetonitrile/water/acetic acid, 79:20:1, *v*/*v*/*v*) was added and the samples were extracted in a vertical position on a GFL3017 rotary shaker (Burgwedel, Germany) for 90 min at room temperature (200 rpm). After extraction, the solid residue was allowed to settle for a few minutes. An aliquot of the raw extract was transferred to an HPLC vial and diluted with the same volume of an acetonitrile/water/acetic acid mixture (20:79:1, *v*/*v*/*v*), resulting in a total dilution factor of 8. Five microlitres of this solution was injected into the UHPLC-MS/MS system without any further cleanup.

### UHPLC-MS/MS parameters

For the analysis, a 1290 Infinity ultra-high-performance liquid chromatography (UHPLC) system coupled to a 6460 Triple Quadrupole mass spectrometer (both Agilent Technologies, Waldbronn, Germany) was used. Chromatographic separation was performed at 25 °C and a flow rate of 250 μL min^−1^ using a ZORBAX Eclipse Plus C18 Rapid Resolution High Definition (150 × 2.1 mm, 1.8 μm) column from Agilent Technologies. Eluent composition was chosen according to Sulyok et al. [4], and the gradient was modified to enhance the separation of the analytes. Hence, the eluents were composed of methanol/water/acetic acid (eluent A—10:89:1, *v*/*v*/*v*; eluent B—97:2:1, *v*/*v*/*v*) containing 5 mM ammonium acetate. The total run time of one chromatographic run was 21 min: after an initial hold time of 2 min at 100 % A, 50 % B was reached within 3 min and 100 % B within the next 9 min. A hold time of 4.5 min at 100 % B was followed by 2.5 min at 100 % A for column re-equilibration. Before injecting the sample into the UHPLC system, the needle was washed in the flush port with acetonitrile/water (50:50, *v*/*v*) for 5 s.

Precursor and product ion selection and the optimisation of fragmentor voltages and collision energies were performed with flow injection of single-analyte solutions using MassHunter Optimizer Triple Quad B04.01. Optimisation was either done in one or in both ionisation modes depending on our experiences with previous methods. MassHunter Data Acquisition software version B04.01 was used to control the LC-MS/MS instrument. Analysis was carried out using electrospray ionisation (ESI) and the dynamic multiple reaction monitoring (DMRM) acquisition. DMRM allows the measurement of selected reaction monitoring transitions for a specified time period (expected retention time ± variable window width), hence resulting in maximized dwell times for each transition. Due to the amount of analytes and to ensure optimum ionisation yields, analysis was performed in two chromatographic runs—one for each ionisation mode. For each compound, two mass transitions were monitored (except for moniliformin and 3-nitropropionic acid), resulting in 4.0 identification points, which is in agreement with Commission Decision 2002/657/EC [29]. The general source settings were as follows: gas temperature, 200 °C; gas flow, 8 L min^−1^; nebulizer, 40 psi (275.8 kPa); sheath gas temperature, 350 °C; sheath gas flow, 11 L min^−1^; capillary voltage, 3,500 V; and nozzle voltage, 500 V (positive) or 0 V (negative). Both scanning quadrupoles (MS1 and MS2) were set to unit resolution. Cycle time was set to 750 ms.

### Method validation

For validation purposes, one blank sample of each commodity (almond, hazelnut, peanut and pistachio) was selected. For each matrix, four times 0.50 ± 0.01 g thoroughly homogenised and ground sample was weighed in 16-mL glass vials. Three of those samples were spiked on one medium concentration level by adding an aliquot of the multi-analyte stock solution prior to extraction. The samples were stored overnight at 40 °C in a Kelvirton® T60120 drying chamber (Heraeus Instruments, Hanau, Germany) to allow evaporation of the solvent. Thereafter, those three spiked samples as well as the fourth blank sample were processed according to the procedure described in “Sample preparation”. The remaining raw extract of the blank sample was used for the preparation of spiked raw extracts on seven levels without replicates and with relative concentrations of 1:3.33:10:33.3:100:333:1,000. This approach (adding the multi-analyte stock solution before and after extraction) allowed the direct determination of the apparent recovery (*R*_A_) as well as the assessment of matrix effects caused by signal suppression or enhancement (SSE) of the analyte signal.

### Data evaluation

For each analyte, linear, 1/*x* weighted calibration curves were calculated by plotting the peak area of the signal in neat standard solution versus the analyte concentration using MassHunter Quantitative Analysis. Apparent recoveries were calculated from the three samples spiked before extraction in the following way: In a first step, the ratio of the measured to spiked concentration for each of the three individual measurements was calculated and multiplied with 100. The average and standard deviation (SD) of those three values was the obtained *R*_A_ and SD for the respective analyte–matrix combination. Matrix effects (SSE) were determined in a similar way as the apparent recoveries, but in this case, the results from the raw extracts of the blank sample spiked on seven different concentration levels after extraction were used for the calculation. Hence, the ratio of the measured to the spiked concentration for these seven levels were calculated and multiplied with 100. For the calculation of the average SSE value, only those levels for which both the qualifier and the quantifier showed a distinct peak were used. The recovery of extraction was calculated by dividing the *R*_A_ value by the SSE value and a multiplication factor of 100. The lower limit of quantification (LLOQ) was calculated as follows: First, the concentration level of the raw extract spiked after extraction for which both mass transitions showed an S/N ratio above 10:1 was determined. This value was then multiplied with the dilution factor of 8 and corrected for the recovery of the extraction step of the respective matrix.

For positive results, the following criteria had to be fulfilled: First, the retention time had to be within ±2.5 % compared to the analyte in neat standard solution and both, the qualifier and the quantifier, transition had to be above an S/N ratio of 10:1. Furthermore, the ion ratio of the quantifier and the qualifier transition had to be within a defined target range according to Commission Decision 2002/657/EC [29]. MassHunter Quantitative Analysis allows setting the criteria for retention time deviation as well as the ratio of quantifier and qualifier; non-compliance is marked in blue (lower) or red (higher) colour. All results obtained for the naturally contaminated samples were multiplied with the dilution factor of 8 and corrected with the apparent recoveries determined during the validation. For the calculation of the average values, all contaminated samples were taken into considerations; for values below the LLOQ, half of the LLOQ of the respective matrix was used.

## Results and discussion

### Development of the analytical method

Based on an HPLC-MS/MS multi-target method developed previously by our group [4, 7], a new UHPLC-MS/MS method was developed on an Agilent 1290 Infinity UHPLC system coupled to a 6460 Triple Quadrupole mass spectrometer. On purpose, we did not speed up the previous method, but used the employed stationary phase with sub-2-μm particles for better resolution of analytes from the matrix. The gradient was adapted from the original method and flattened to allow better separation of the analytes. Another major advantage of this method proved to be the use of the DMRM mode. Early multi-target methods for the determination of mycotoxins in food dealing with a large number of analytes (e.g. [7]) employed time periods for which only certain analytes are measured to allow enough dwell time for each analyte. These periods are prone to retention time shifts due to various reasons, e.g. caused by slightly different solvent compositions, by declining column performances or slight temperature shifts. Using DMRM, the size of the retention time window for each mass transition can be set individually. As chromatographic conditions were very repeatable during method development, we generally set the time window to 1 min. Moreover, for broad chromatographic peaks (e.g. HC toxin and ustiloxin B), window widths of 2 min were used. If two analytes share the same mass transitions, window widths which includes the different retention times can be selected (e.g. 1.5 min for cytochalasin C and D or enniatin B2 and K1). Using these variable window widths, we obtained minimum dwell times of at least 10 ms, even in the most crowded sections of the chromatogram. In order to gain enough points over a given chromatographic peak, we set the duty cycle of the mass spectrometer to 750 ms. The usual peak showed a base width of 0.2 min, resulting in 16 data points, which were well suited for reliable integration of the peak area. Moreover, also cyclopiazonic acid, which is known to give broad peaks under acidic RP-HPLC conditions, showed an acceptable peak width of about 0.5 min in our case.

To ensure optimum sensitivity, we decided to apply two chromatographic runs per sample—one for each ionisation mode. For each analyte, the mode resulting in the most abundant signal was used and two mass transitions were selected: one for the use as quantifier and one as qualifier. The formation of sodium adducts of certain analytes led to insufficient fragment intensity because the positive charge remains on the sodium ion after collision-induced dissociation. Therefore, ammonium acetate was added to both of the solvents. Most analytes were detected as single-charged ions either as protonated [M+H]^+^ or ammonium adducts [M + NH_4_]^+^ (e.g. 4,15-diacetoxyscirpenol, enniatins and mycophenolic acid) in positive mode or as deprotonated [M-H]^−^ or acetate adducts [M+OAc]^−^ (e.g. the B-trichothecenes DON and nivalenol) in negative mode. Exceptions were cyclosporin A, C, D and H which were measured as doubly charged [M+2H]^2+^ ions. For alamethicin F30 (cleavage of the peptide bond) [30] and also for fusarielin A (loss of water) in-source fragments were used as precursors as these ions were predominantly formed using ESI.

Table 1 summarises the obtained retention times, precursor and product ions including the optimised ESI-MS/MS settings for all analytes ionising in negative electrospray mode. The same parameters are given in Table 2 for the analytes showing higher ionisation abundance in positive electrospray mode. Only one fragment ion of sufficient sensitivity was obtained for the very small molecules moniliformin (98 g mol^−1^) and 3-nitropropionic acid (119 g mol^−1^). In general, the most abundant mass transition was used as the quantifier, but when the S/N ratio was significantly worse for this mass transition, another one was used. Using MassHunter Quantitative Analysis, the qualifier-to-quantifier ratios based on the peak area were calculated and non-compliant values according to Commission Decision 2002/657/EC [29] were flagged automatically. Compared to a previously described method for the determination of 186 analytes including 164 fungal metabolites in dust [30], we describe a method for the determination of a total number of 191 fungal metabolites which include all relevant mycotoxins. Bacterial metabolites, which can occur in house dust, were eliminated from the former method as those metabolites are not relevant for food.

**Table 1:** List of analytes determined in the negative ionisation mode and optimised ESI-MS/MS parameters

Analyte name	Retention time (min)	*m*/*z* precursor ion (framentor voltage)	Ion species	*m*/*z* product ion (collision energy (V))
3-Acetyldeoxynivalenol	7.4	397 (95)	[M+OAc]^−^	307 (8)/59 (20)
alpha-Zearalenol	11.8	319 (195)	[M-H]^−^	160 (28)/130 (32)
alpha-Zearalenol-14-glucoside	9.2	541 (100)	[M+OAc]^−^	319 (16)/481 (0)
Alternariol	10.4	257 (190)	[M-H]^−^	213 (16)/215 (20)
Alternariolmethylether	12.5	271 (170)	[M-H]^−^	256 (16)/227 (32)
Altersolanol C	7.8	319 (105)	[M-H]^−^	283 (12)/301 (8)
Altertoxin I	10.0	351 (140)	[M-H]^−^	315 (8)/263 (32)
Apicidin	13.0	622 (220)	[M-H]^−^	462 (16)/252 (32)
Atpenin A5	13.5	364 (120)	[M-H]^−^	292 (4)/328 (0)
beta-Zearalenol	10.7	319 (185)	[M-H]^−^	160 (28)/130 (32)
beta-Zearalenol-14-glucoside	8.1	541 (115)	[M+OAc]^−^	319 (16)/481 (0)
Cycloechinulin	9.3	350 (170)	[M-H]^−^	335 (20)/320 (28)
Cyclopiazonic acid	12.2	335 (270)	[M-H]^−^	140 (28)/154 (32)
Deoxynivalenol	5.9	355 (95)	[M+OAc]^−^	265 (8)/59 (16)
Deoxynivalenol-3-glucoside	5.8	517 (145)	[M+OAc]^−^	427 (16)/457 (8)
Emodin	14.1	269 (185)	[M-H]^−^	225 (20)/241 (20)
Equisetin	15.2	372 (205)	[M-H]^−^	342 (20)/124 (48)
Fusarenon-X	6.6	413 (110)	[M+OAc]^−^	263 (8)/59 (28)
Fusidic acid	15.1	515 (190)	[M-H]^−^	221 (24)/455 (16)
Macrosporin	13.6	283 (170)	[M-H]^−^	268 (16)/225 (36)
Moniliformin	1.5	97 (65)	[M-H]^−^	41 (8)
Nidulin	15.4	441 (185)	[M-H]^−^	382 (20)/390 (24)
3-Nitropropionic acid	2.2	118 (65)	[M-H]^−^	46 (8)
Nivalenol	4.8	371 (110)	[M+OAc]^−^	281 (8)/59 (20)
Nornidulin	14.1	427 (160)	[M-H]^−^	347 (16)/376 (20)
Patulin	4.5	153 (70)	[M-H]^−^	109 (4)/81 (4)
Penigequinolone A	13.0	466 (200)	[M-H]^−^	394 (32)/378 (48)
Physcion	15.4	283 (145)	[M-H]^−^	240 (20)/211.6 (40)
Pseurotin A	8.6	430 (100)	[M-H]^−^	270 (4)/308 (0)
Radicicol	9.0	363 (165)	[M-H]^−^	183 (20)/224 (16)
Rubellin D	12.7	541 (165)	[M-H]^−^	360 (24)/378 (16)
Tentoxin	10.4	413 (160)	[M-H]^−^	271 (12)/141 (12)
Tenuazonic acid	8.4	196 (150)	[M-H]^−^	112 (20)/139 (16)
Zearalenone	11.9	317 (195)	[M-H]^−^	131 (28)/175 (20)
Zearalenone-14-glucoside	9.4	479 (190)	[M-H]^−^	317 (8)/175 (44)
Zearalenone-14-sulphate	8.8	397 (160)	[M-H]^−^	317 (20)/131 (44)

**Table 2:** List of analytes determined in the positive ionisation mode and optimised ESI-MS/MS parameters

Analyte name	Retention time (min)	*m*/*z* precursor ion (framentor voltage)	Ion species	*m*/*z* product ion (collision energy (V))
AAL toxin TA 1	9.0	522 (165)	[M+H]^+^	328.5 (24)/292.4 (28)
15-Acetyldeoxynivalenol	7.4	339 (110)	[M+H]^+^	321 (4)/137 (4)
Aflatoxin B_1_	8.7	313 (165)	[M+H]^+^	285.2 (20)/128.1 (70)
Aflatoxin B_2_	8.4	315 (190)	[M+H]^+^	287 (24)/259 (28)
Aflatoxin G_1_	8.0	329 (175)	[M+H]^+^	200 (44)/243.2 (24)
Aflatoxin G_2_	7.7	331 (190)	[M+H]^+^	313 (24)/245 (28)
Aflatoxin M_1_	7.7	329 (180)	[M+H]^+^	273 (24)/229 (28)
Aflatoxin M_2_	7.3	331 (145)	[M+H]^+^	273 (20)/285 (20)
Agroclavine	6.9	239 (130)	[M+H]^+^	183 (16)/208 (16)
Alamethicin F30	14.9	775 (225)	[y7^a^+H]^+^	282 (44)/197 (52)
Altenuene	8.6	293 (100)	[M+H]^+^	257 (8)/275 (4)
Altenusin	8.8	291 (90)	[M+H]^+^	128 (56)/199 (32)
2-Amino-14,16-dimethyloctadecan-3-ol	15.5	314 (115)	[M+H]^+^	296.5 (16)/125 (12)
Aspercolorin	10.9	465 (155)	[M+H]^+^	247 (16)/120 (48)
Aspergillimide	6.8	360 (230)	[M+H]^+^	301 (36)/332 (12)
Asperlactone	5.6	185 (65)	[M+H]^+^	141 (0)/113 (4)
Asperloxin A	10.1	394 (205)	[M+H]^+^	123 (20)/95 (44)
Aspinonene	4.9	206 (80)	[M+NH_4_]^+^	127 (0)/81 (12)
Aspyrone	6.2	185 (75)	[M+H]^+^	125 (4)/139 (0)
Asterric acid	9.5	349 (80)	[M+H]^+^	299 (8)/287 (12)
Aureobasidin A	15.8	1102 (270)	[M+H]^+^	665.5 (28)/210 (48)
Austdiol	6.5	237 (125)	[M+H]^+^	117 (28)/159 (20)
Austocystin A	13.1	373 (165)	[M+H]^+^	329 (28)/312 (28)
Avenacein Y	10.0	319 (120)	[M+H]^+^	175 (36)/287 (16)
Beauvericin	15.0	801.5 (180)	[M+NH_4_]^+^	244 (36)/262 (32)
Brefeldin A	10.2	281 (75)	[M+H]^+^	245 (0)/199 (4)
Brevicompanine B	12.6	368 (120)	[M+H]^+^	130 (24)/300 (8)
Calphostin C	13.9	791 (220)	[M+H]^+^	485 (32)/515 (20)
Cephalosporin C	4.0	416 (143)	[M+H]^+^	185 (12)/143 (12)
Cerulenin	9.7	224 (85)	[M+H]^+^	196 (0)/179 (0)
Chaetocin	11.5	697 (135)	[M+H]^+^	348 (12)/350 (12)
Chaetoglobosin A	12.3	529 (135)	[M+H]^+^	130 (44)/511 (4)
Chaetomin	12.2	711 (155)	[M+H]^+^	298 (12)/348 (8)
Chanoclavine	6.3	257 (105)	[M+H]^+^	168 (16)/226 (8)
Chlamydosporol	7.1	227 (110)	[M+H]^+^	167 (12)/106 (32)
Citreoviridin A	11.9	420 (95)	[M+NH_4_]^+^	315.4 (0)/285 (8)
Citrinin	8.9	251 (120)	[M+H]^+^	233 (12)/205 (28)
Citromycetin	8.7	291 (115)	[M+H]^+^	245 (24)/217 (32)
Cochliodinol	16.0	507 (150)	[M+H]^+^	371 (20)/439 (8)
Curvularin	9.7	310 (75)	[M+NH_4_]^+^	125 (12)/169 (8)
Cyclopenin	8.5	295 (105)	[M+H]^+^	146 (20)/177 (8)
Cyclopeptine	9.4	281 (140)	[M+H]^+^	120 (20)/134 (20)
Cyclosporin A+H^b^	16.0	602 (140)	[M+2H]^2+^	100 (56)/156 (32)
Cyclosporin C	15.4	610 (125)	[M+2H]^2+^	100 (52)/156 (32)
Cyclosporin D	16.3	609 (140)	[M+2H]^2+^	100 (52)/156 (32)
Cytochalasin A	12.7	478 (190)	[M+H]^+^	91 (60)/120 (24)
Cytochalasin B	10.5	480 (160)	[M+H]^+^	462.5 (12)/444.5 (12)
Cytochalasin C	11.2	525 (110)	[M+NH_4_]^+^	430.5 (12)/490.5 (4)
Cytochalasin D	10.4	525 (110)	[M+NH_4_]^+^	430.5 (12)/490.5 (4)
Cytochalasin E	11.6	513 (110)	[M+NH_4_]^+^	434.5 (4)/416 (4)
Cytochalasin H	10.6	494 (135)	[M+H]^+^	434.5 (4)/416.5 (8)
Cytochalasin J	9.8	452 (145)	[M+H]^+^	434.5 (4)/416.5 (12)
Decarestrictine D	5.5	217 (80)	[M+H]^+^	121 (8)/163 (4)
Dechlorogriseofulvin	9.1	319 (135)	[M+H]^+^	181 (12)/251 (16)
Deoxybrevianamid E	10.1	352 (110)	[M+H]^+^	130 (28)/284 (8)
4,15-Diacetoxyscirpenol	8.7	384 (110)	[M+NH_4_]^+^	307 (4)/247 (8)
Dihydroergine	5.5	270 (150)	[M+H]^+^	168 (28)/210 (20)
Dihydroergosine	8.3	550 (190)	[M+H]^+^	270 (28)/253 (28)
Dihydroergotamine	8.5	584 (190)	[M+H]^+^	270 (32)/254 (32)
Dihydrolysergol	6.0	257 (160)	[M+H]^+^	167 (44)/154 (40)
Elymoclavine	5.9	255 (135)	[M+H]^+^	181 (28)/180 (44)
Elymoclavine-fructoside	5.5	417 (165)	[M+H]^+^	255 (16)/237 (20)
Enniatin A	15.5	699 (170)	[M+NH_4_]^+^	210 (32)/228 (32)
Enniatin A1	15.3	685 (150)	[M+NH_4_]^+^	210 (32)/228 (32)
Enniatin B	14.7	657 (160)	[M+NH_4_]^+^	196 (32)/214 (32)
Enniatin B1	15.0	672 (170)	[M+NH_4_]^+^	196 (32)/100 (60)
Enniatin B2	14.1	643 (145)	[M+NH_4_]^+^	196 (28)/214 (28)
Enniatin B3	13.8	629 (145)	[M+NH_4_]^+^	196 (28)/214 (28)
Enniatin B4	14.7	671.5 (170)	[M+NH_4_]^+^	196 (32)/100 (60)
Enniatin K1	14.4	643 (145)	[M+NH_4_]^+^	196 (28)/214 (28)
Ergine+Erginine^b^	5.5	268 (125)	[M+H]^+^	223 (16)/208 (24)
Ergocornine	8.6	562 (165)	[M+H]^+^	208 (48)/223 (36)
Ergocorninine	10.0	562 (150)	[M+H]^+^	277 (24)/223 (36)
Ergocristine	9.3	610 (140)	[M+H]^+^	592.5 (12)/223 (36)
Ergocristinine	11.0	610 (140)	[M+H]^+^	592.5 (12)/223 (36)
Ergocryptine	9.3	576 (155)	[M+H]^+^	208 (52)/223 (36)
Ergocryptinine	10.7	576 (155)	[M+H]^+^	558.6 (12)/223 (36)
Ergometrine	5.9	326 (145)	[M+H]^+^	223 (20)/208 (28)
Ergometrinine	6.5	326 (145)	[M+H]^+^	223 (20)/208 (28)
Ergosine	8.2	548 (165)	[M+H]^+^	223 (32)/208 (44)
Ergosinine	9.5	548 (165)	[M+H]^+^	530.5 (12)/223 (32)
Ergotamine+Ergotaminine^b^	8.5	582 (155)	[M+H]^+^	223 (32)/208 (48)
Festuclavine	7.0	241 (160)	[M+H]^+^	154 (36)/168 (28)
Fulvic acid	7.6	309 (115)	[M+H]^+^	231 (20)/161 (40)
Fumagillin	13.0	459 (140)	[M+H]^+^	177 (8)/131 (24)
Fumigaclavin A	6.6	299 (170)	[M+H]^+^	167 (48)/154 (44)
Fumitremorgin C	10.7	380 (145)	[M+H]^+^	226 (16)/212 (32)
Fumonisin B_1_	10.3	722.5 (210)	[M+H]^+^	352.3 (36)/334.4 (44)
Fumonisin B_2_	12.1	706.3 (220)	[M+H]^+^	336.3 (40)/318.5 (40)
Fumonisin B_3_	11.3	706.5 (220)	[M+H]^+^	336 (40)/318.5 (40)
Fusaproliferin	14.6	445 (105)	[M+H]^+^	385 (4)/367 (4)
Fusarielin A	15.1	385 (130)	[M-H_2_O+H]^+^	109 (16)/253 (8)
Geodin	11.4	399 (125)	[M+H]^+^	340 (20)/355 (4)
Gibberellic acid	7.0	364 (105)	[M+NH_4_]^+^	329 (4)/221 (24)
Gliotoxin	8.8	327 (95)	[M+H]^+^	263 (4)/245 (16)
Griseofulvin	9.8	353 (140)	[M+H]^+^	165 (16)/215 (16)
HC toxin	7.5	437 (150)	[M+H]^+^	169 (28)/240 (16)
HT-2 toxin	10.0	442.2 (100)	[M+NH_4_]^+^	263.1 (8)/215 (4)
Hydrolysed fumonisin B_1_	9.7	406 (150)	[M+H]^+^	388 (12)/370 (16)
16-Keto-aspergillimide	13.1	374 (185)	[M+H]^+^	313 (36)/315 (28)
Kojic acid	2.5	143 (120)	[M+H]^+^	69 (16)/97 (12)
Lysergol	6.0	255 (130)	[M+H]^+^	240 (20)/197 (20)
Malformin C	12.6	530 (205)	[M+H]^+^	417 (12)/372 (16)
Marcfortine A	9.0	478 (170)	[M+H]^+^	450 (16)/419 (32)
Meleagrin	8.8	434 (140)	[M+H]^+^	403 (12)/334 (20)
3-*O*-Methylsterigmatocystin	11.0	339 (160)	[M+H]^+^	306 (28)/324 (20)
3-*O*-Methylviridicatin	11.3	252 (155)	[M+H]^+^	236 (28)/190 (48)
Mevastatin	13.9	408 (95)	[M+NH_4_]^+^	185 (12)/271 (8)
Mevinolin	14.3	422 (110)	[M+NH_4_]^+^	199 (4)/173 (24)
15-Monoacetoxyscirpenol	7.9	342 (95)	[M+NH_4_]^+^	265 (0)/307 (4)
Mycophenolic acid	10.7	338 (75)	[M+NH_4_]^+^	207 (28)/303 (8)
Neosolaniol	6.6	400 (110)	[M+NH_4_]^+^	185 (16)/215 (12)
Neoxaline	8.4	436 (130)	[M+H]^+^	405 (8)/263 (36)
Ochratoxin A	11.5	404 (115)	[M+H]^+^	239 (20)/102 (70)
Ochratoxin B	10.3	370 (115)	[M+H]^+^	205 (16)/103 (64)
Ophiobolin A	13.2	401 (110)	[M+H]^+^	365 (4)/267 (8)
Ophiobolin B	13.8	403 (105)	[M+H]^+^	367 (4)/349 (12)
Oxaspirodione	7.9	251 (110)	[M+H]^+^	133 (16)/161 (4)
Paraherquamide A	8.4	494 (160)	[M+H]^+^	419 (32)/176 (44)
Paspalic acid	5.5	269 (140)	[M+H]^+^	182 (28)/167 (44)
Paxilline	14.1	436 (120)	[M+H]^+^	182 (32)/167 (76)
Penicillic acid	6.6	171 (70)	[M+H]^+^	125 (8)/97 (12)
Penicillin G	6.8	335 (95)	[M+H]^+^	160 (4)/176 (8)
Penitrem A	13.9	634 (165)	[M+H]^+^	558 (16)/616 (4)
Pentoxyfylline	7.3	279 (130)	[M+H]^+^	181 (16)/99 (16)
Pestalotin	8.5	215 (90)	[M+H]^+^	85 (12)/153 (8)
Phomopsin A	7.3	789 (205)	[M+H]^+^	226 (40)/323 (24)
Phomopsin B	7.0	755 (165)	[M+H]^+^	192 (40)/289 (24)
Pyripyropene A	11.6	584 (260)	[M+H]^+^	148 (56)/202 (36)
Roquefortine C	10.2	390 (145)	[M+H]^+^	193 (24)/322 (16)
Roridin A	11.2	550 (120)	[M+NH_4_]^+^	249 (12)/231 (20)
Rugulosin	12.4	543 (145)	[M+H]^+^	273 (20)/255 (36)
Secalonic acid D	13.3	639 (220)	[M+H]^+^	561.4 (24)/589.4 (24)
Setosusin	10.5	532 (160)	[M+NH_4_]^+^	413 (20)/299 (28)
Sterigmatocystin	12.3	325 (160)	[M+H]^+^	281 (40)/130 (24)
Sulochrin	8.3	333 (95)	[M+H]^+^	209 (4)/136 (48)
T-2 tetraol	4.8	316 (95)	[M+NH_4_]^+^	215 (4)/281 (4)
T-2 toxin	10.9	484.3 (120)	[M+NH_4_]^+^	185.1 (16)/215.2 (12)
T-2 triol	9.1	400 (105)	[M+NH_4_]^+^	215 (4)/281 (0)
Terphenyllin	9.1	339 (125)	[M+H]^+^	307 (8)/292 (20)
Territrem B	11.7	527 (205)	[M+H]^+^	291 (28)/491 (20)
Trichodermin	11.1	293 (100)	[M+H]^+^	109 (16)/143 (12)
Tryprostatin A	10.2	382 (115)	[M+H]^+^	326 (8)/228 (12)
Ustiloxin A	5.5	674 (220)	[M+H]^+^	187 (32)/209 (36)
Ustiloxin B	4.7	646.2 (170)	[M+H]^+^	181 (36)/187 (28)
Ustiloxin D	5.7	495 (135)	[M+H]^+^	192 (20)/291 (12)
Verrucarin A	10.9	520 (125)	[M+NH_4_]^+^	249 (12)/457 (8)
Verrucofortine	11.9	410 (150)	[M+H]^+^	130 (32)/300 (16)
Verruculogen	12.8	512 (125)	[M+H]^+^	352 (16)/494 (0)
Viomellein	12.5	561 (215)	[M+H]^+^	530 (28)/511 (32)
Viridicatin	11.2	238 (155)	[M+H]^+^	165 (36)/192 (24)
Wortmannin	8.8	429 (95)	[M+H]^+^	355 (4)/295 (20)

^a^In-source fragment obtained from the cleavage of the corresponding peptide bond

^b^Due to co-elution and the same DMRM transitions, these analytes cannot be distinguished

### Method performance parameters

Method performance parameters were obtained by spiking blank samples before extraction on one medium level in triplicate and the raw extract of a blank sample after extraction on seven levels without replicates. It has to be pointed out that only 0.5 g of highly homogenised blank nuts was used for validation purposes to minimise the amount of spiked toxins. The results are presented in Tables 3, 4, 5 and 6 for almonds, hazelnuts, peanuts and pistachios, respectively. Spiking was performed with the whole multi-analyte mix containing all metabolites, but data evaluation was restricted to the most important analytes, e.g. detected in the naturally contaminated nut samples, regulated mycotoxins or mycotoxins frequently found in other commodities. In total, method performance parameters for 65 analytes are shown and are discussed in the following. For about half of the analytes, apparent recoveries between 80 and 120 % were achieved. This number was expected since a multi-target method covering a huge number of chemically diverse analytes always has to be a compromise. The chosen extraction solvent, as well as chromatographic conditions (regarding separation from matrix), was a compromise to include all analytes into the method. For example, in the case of ergot alkaloids, epimerisation is favoured under the used acidic conditions and, hence, only a rough estimate of a sum concentration is possible [31]. For the determination of specific analytes at a very high level of sensitivity, more dedicated methods (validated for the respective matrix) are recommended. These methods often also allow sample concentration as cleanup procedures can be employed. For the accurate determination of regulated mycotoxins, the use of stable isotope-labelled internal standards is also an excellent option. For instance, deuterated AFB_2_ and AFG_2_ had been used for the quantification of aflatoxins in almonds [32] or 11 U-[^13^C]-labelled mycotoxins were applied in the analysis of maize [33].

**Table 3:** Method performance characteristics determined for almonds

Analyte	LLOQ (μg kg^−1^)	*R*_A_ (%) ± SD^a^	SSE (%)^b^	*R*_E_ (%)^c^
3-Acetyldeoxynivalenol	48	51 ± 22	45	112
Aflatoxin B_1_	3.0	101 ± 6	87	115
Aflatoxin B_2_	10	81 ± 10	81	100
Aflatoxin G_1_	10	78 ± 1	78	101
Aflatoxin G_2_	8.2	85 ± 1	67	127
Aflatoxin M_1_	7.9	73 ± 3	70	104
Alamethicin F30	190	33 ± 5	88	38
alpha-Zearalenol	16	125 ± 7	137	91
alpha-Zearalenol-14-glucoside	110	98 ± 10	126	78
Alternariol	3.0	115 ± 5	126	91
Alternariolmethylether	0.8	117 ± 7	108	109
Altertoxin I	13	95 ± 4	104	91
Apidicin	0.7	98 ± 4	117	84
Atpenin A5	1.3	124 ± 8	123	101
Beauvericin	0.2	86 ± 8	85	102
beta-Zearalenol	15	143 ± 2	145	98
beta-Zearalenol-14-glucoside	99	68 ± 5	79	87
Chaetocin	110	29 ± 1	126	23
Chlamydosporol	53	101 ± 4	78	129
Curvularin	31	107 ± 10	105	101
Cycloechinulin	1.0	195 ± 12	187	104
Cyclopiazonic acid	60	124 ± 13	166	75
Cyclosporin A+H	29	66 ± 4	70	95
Cytochalasin J	25	96 ± 6	94	101
Deoxynivalenol	470	22 ± 4	19	116
4,15-Diacetoxyscirpenol	16	104 ± 8	92	114
Emodin	3.4	120 ± 9	150	80
Enniatin A	2.4	106 ± 23	81	132
Enniatin A1	2.0	76 ± 18	77	99
Enniatin B	0.1	100 ± 11	107	93
Enniatin B1	0.2	84 ± 8	83	102
Enniatin B2	0.7	107 ± 11	99	108
Enniatin B3	0.05	108 ± 8	96	113
Enniatin B4	0.1	88 ± 8	99	89
Enniatin K1	0.3	95 ± 5	97	98
Equisetin	17	222 ± 11	285	78
Fumonisin B_1_	160	41 ± 4	100	42
Fumonisin B_2_	180	38 ± 7	96	39
HC toxin	130	35 ± 9	58	60
HT-2 toxin	46	113 ± 8	95	119
Kojic acid	230	34 ± 2	38	89
Macrosporin	2.7	165 ± 20	146	113
Meleagrin	21	104 ± 9	95	109
3-*O*-Methylsterigmatocystin	2.0	99 ± 5	97	102
Moniliformin	5.1	15 ± 1	17	88
Mycophenolic acid	24	101 ± 8	94	107
Neoxaline	3.0	93 ± 7	103	90
Nidulin	0.5	73 ± 3	99	74
3-Nitropropionic acid	5.8	45 ± 4	46	98
Nivalenol	44	14 ± 3	12	123
Nornidulin	1.2	111 ± 8	125	88
Ochratoxin A	15	90 ± 8	89	101
Ochratoxin B	9.9	84 ± 5	94	89
Penigequinolone A	0.4	142 ± 14	150	95
Pestalotin	6.0	112 ± 11	98	114
Physcion	260	155 ± 13	293	53
Pseurotin A	25	159 ± 12	173	92
Roquefortine C	5.5	136 ± 7	97	141
Secalonic acid D	41	77 ± 5	99	78
Sterigmatocystin	2.8	106 ± 7	108	98
T-2 toxin	5.2	105 ± 9	101	104
Tentoxin	1.2	111 ± 4	120	92
Viridicatin	7.5	104 ± 7	100	104
Zearalenone	5.3	127 ± 6	122	104
Zearalenone-14-sulphate	5.2	62 ± 6	86	72

*LLOQ* lower limit of quantification

^a^Apparent recovery and standard deviation thereof (triplicate analysis on medium level)

^b^Mean value for the signal suppression or enhancement calculated from up to seven concentration levels without replicates

^c^Calculated extraction recovery (*R*_A_/SSE × 100)

**Table 4:** Method performance characteristics determined for hazelnuts

Analyte	LLOQ (μg kg^−1^)	*R*_A_ (%) ± SD^a^	SSE (%)^b^	*R*_E_ (%)^c^
3-Acetyldeoxynivalenol	54	55 ± 6	55	100
Aflatoxin B_1_	10	97 ± 13	98	99
Aflatoxin B_2_	11	87 ± 5	90	97
Aflatoxin G_1_	4.4	81 ± 6	103	79
Aflatoxin G_2_	11	83 ± 8	89	93
Aflatoxin M_1_	9.8	68 ± 7	81	84
Alamethicin F30	210	31 ± 7	90	35
alpha-Zearalenol	18	99 ± 12	122	81
alpha-Zearalenol-14-glucoside	490	71 ± 9	121	58
Alternariol	3.0	95 ± 7	107	89
Alternariolmethylether	2.9	95 ± 7	101	94
Altertoxin I	14	90 ± 5	106	85
Apidicin	0.8	77 ± 9	113	69
Atpenin A5	1.6	104 ± 10	127	82
Beauvericin	0.6	77 ± 3	89	87
beta-Zearalenol	16	106 ± 8	118	89
beta-Zearalenol-14-glucoside	170	64 ± 6	127	51
Chaetocin	180	23 ± 1	158	14
Chlamydosporol	69	107 ± 3	107	100
Curvularin	33	97 ± 3	105	93
Cycloechinulin	3.6	132 ± 13	138	96
Cyclopiazonic acid	56	121 ± 23	149	81
Cyclosporin A+H	36	57 ± 7	73	78
Cytochalasin J	34	77 ± 4	101	76
Deoxynivalenol	46	40 ± 8	34	118
4,15-Diacetoxyscirpenol	19	97 ± 6	101	96
Emodin	3.9	99 ± 9	143	70
Enniatin A	3.0	73 ± 20	72	102
Enniatin A1	0.5	83 ± 6	73	114
Enniatin B	0.1	99 ± 8	104	95
Enniatin B1	0.2	78 ± 7	82	95
Enniatin B2	1.0	82 ± 7	105	79
Enniatin B3	0.1	92 ± 6	100	92
Enniatin B4	0.1	75 ± 1	104	72
Enniatin K1	0.5	74 ± 2	107	69
Equisetin	17	188 ± 15	242	78
Fumonisin B_1_	190	37 ± 5	105	35
Fumonisin B_2_	240	31 ± 3	109	29
HC toxin	500	31 ± 7	61	51
HT-2 toxin	59	94 ± 7	100	93
Kojic acid	280	34 ± 3	47	73
Macrosporin	3.2	146 ± 25	150	98
Meleagrin	19	95 ± 4	79	120
3-*O*-Methylsterigmatocystin	2.4	89 ± 3	103	86
Moniliformin	5.3	14 ± 1	17	84
Mycophenolic acid	27	93 ± 6	100	92
Neoxaline	3.5	97 ± 10	128	76
Nidulin	0.6	61 ± 4	103	60
3-Nitropropionic acid	7.1	36 ± 2	45	79
Nivalenol	86	16 ± 1	26	63
Nornidulin	0.5	93 ± 12	143	65
Ochratoxin A	18	73 ± 7	92	80
Ochratoxin B	14	67 ± 3	108	62
Penigequinolone A	0.5	116 ± 20	157	74
Pestalotin	6.2	109 ± 3	98	111
Physcion	270	129 ± 6	252	51
Pseurotin A	3.0	111 ± 19	143	78
Roquefortine C	6.0	119 ± 9	93	128
Secalonic acid D	49	69 ± 10	106	65
Sterigmatocystin	3.1	87 ± 3	99	88
T-2 toxin	5.6	90 ± 7	93	96
Tentoxin	1.2	93 ± 12	107	87
Viridicatin	8.3	96 ± 5	103	94
Zearalenone	2.2	91 ± 7	111	82
Zearalenone-14-sulphate	7.8	47 ± 5	100	47

*LLOQ* lower limit of quantification

^a^Apparent recovery and standard deviation thereof (triplicate analysis on medium level)

^b^Mean value for the signal suppression or enhancement calculated from up to seven concentration levels without replicates

^c^Calculated extraction recovery (*R*_A_/SSE × 100)

**Table 5:** Method performance characteristics determined for peanuts

Analyte	LLOQ (μg kg^−1^)	R_A_ (%) ± SD^a^	SSE (%)^b^	*R*_E_ (%)^c^
3-Acetyldeoxynivalenol	190	17 ± 1	17	96
Aflatoxin B_1_	10	84 ± 9	85	99
Aflatoxin B_2_	10	84 ± 4	82	103
Aflatoxin G_1_	12	67 ± 5	75	88
Aflatoxin G_2_	9.4	73 ± 4	66	111
Aflatoxin M_1_	8.7	65 ± 3	69	95
Alamethicin F30	110	63 ± 4	91	69
alpha-Zearalenol	14	107 ± 2	108	99
alpha-Zearalenol-14-glucoside	97	104 ± 2	117	88
Alternariol	8.8	101 ± 3	98	102
Alternariolmethylether	2.7	84 ± 1	84	101
Altertoxin I	14	79 ± 2	87	90
Apidicin	0.6	97 ± 3	107	91
Atpenin A5	1.2	132 ± 3	126	105
Beauvericin	0.4	94 ± 8	82	115
beta-Zearalenol	15	115 ± 2	119	97
beta-Zearalenol-14-glucoside	99	46 ± 2	54	87
Chaetocin	120	24 ± 1	114	21
Chlamydosporol	16	125 ± 9	97	129
Curvularin	29	107 ± 4	100	107
Cycloechinulin	3.1	148 ± 5	135	110
Cyclopiazonic acid	61	84 ± 2	114	74
Cyclosporin A+H	28	64 ± 8	64	99
Cytochalasin J	26	92 ± 4	92	101
Deoxynivalenol	150	29 ± 1	24	120
4,15-Diacetoxyscirpenol	18	92 ± 4	91	100
Emodin	11	122 ± 6	151	81
Enniatin A	2.8	86 ± 17	79	110
Enniatin A1	1.4	101 ± 37	72	141
Enniatin B	0.05	117 ± 16	81	144
Enniatin B1	0.1	102 ± 8	89	114
Enniatin B2	2.4	95 ± 11	87	109
Enniatin B3	0.04	100 ± 3	79	126
Enniatin B4	0.2	97 ± 4	92	106
Enniatin K1	0.3	98 ± 8	88	111
Equisetin	13	267 ± 13	276	97
Fumonisin B_1_	210	33 ± 8	102	33
Fumonisin B_2_	180	40 ± 3	105	38
HC toxin	130	18 ± 1	30	58
HT-2 toxin	50	99 ± 8	90	110
Kojic acid	280	27 ± 1	37	74
Macrosporin	2.6	166 ± 6	137	121
Meleagrin	16	107 ± 2	73	146
3-*O*-Methylsterigmatocystin	1.9	88 ± 2	82	106
Moniliformin	6.3	16 ± 1	23	72
Mycophenolic acid	26	84 ± 3	87	97
Neoxaline	2.8	113 ± 4	119	95
Nidulin	0.5	75 ± 1	98	76
3-Nitropropionic acid	6.9	51 ± 1	62	82
Nivalenol	69	19 ± 1	25	78
Nornidulin	1.2	118 ± 1	137	86
Ochratoxin A	17	78 ± 3	89	88
Ochratoxin B	37	77 ± 5	96	80
Penigequinolone A	1.0	146 ± 5	139	105
Pestalotin	6.2	101 ± 5	91	111
Physcion	260	161 ± 12	295	54
Pseurotin A	23	152 ± 9	150	102
Roquefortine C	5.2	94 ± 5	63	149
Secalonic acid D	38	105 ± 5	125	84
Sterigmatocystin	2.4	97 ± 3	84	115
T-2 toxin	5.1	94 ± 7	88	107
Tentoxin	1.1	108 ± 5	115	93
Viridicatin	7.8	95 ± 2	96	100
Zearalenone	5.7	109 ± 1	112	97
Zearalenone-14-sulphate	5.2	65 ± 2	94	69

*LLOQ* lower limit of quantification

^a^Apparent recovery and standard deviation thereof (triplicate analysis on medium level)

^b^Mean value for the signal suppression or enhancement calculated from up to seven concentration levels without replicates

^c^Calculated extraction recovery (*R*_A_/SSE × 100)

**Table 6:** Method performance characteristics determined for pistachios

Analyte	LLOQ (μg kg^−1^)	*R*_A_ (%) ± SD^a^	SSE (%)^b^	*R*_E_ (%)^c^
3-Acetyldeoxynivalenol	45	37 ± 5	31	120
Aflatoxin B_1_	10	85 ± 6	83	102
Aflatoxin B_2_	11	42 ± 3	43	96
Aflatoxin G_1_	10	69 ± 6	69	100
Aflatoxin G_2_	11	62 ± 11	64	97
Aflatoxin M_1_	8.9	60 ± 6	65	93
Alamethicin F30	243	28 ± 3	94	30
alpha-Zearalenol	17	100 ± 2	117	85
alpha-Zearalenol-14-glucoside	100	67 ± 5	80	83
Alternariol	9.6	97 ± 1	102	94
Alternariolmethylether	0.9	107 ± 2	107	100
Altertoxin I	14	91 ± 2	101	90
Apidicin	0.7	82 ± 2	110	74
Atpenin A5	1.4	121 ± 3	131	92
Beauvericin	0.6	72 ± 10	78	92
beta-Zearalenol	15	119 ± 7	126	95
beta-Zearalenol-14-glucoside	120	72 ± 1	97	74
Chaetocin	260	33 ± 1	112	29
Chlamydosporol	200	83 ± 3	82	101
Curvularin	100	100 ± 8	100	100
Cycloechinulin	1.1	129 ± 7	134	96
Cyclopiazonic acid	58	131 ± 6	168	78
Cyclosporin A+H	36	59 ± 1	75	79
Cytochalasin J	28	85 ± 8	91	93
Deoxynivalenol	241	18 ± 4	8	225
4,15-Diacetoxyscirpenol	5.4	92 ± 4	91	102
Emodin	3.5	114 ± 2	147	77
Enniatin A	2.5	95 ± 13	76	126
Enniatin A1	2.3	65 ± 4	77	84
Enniatin B	0.1	105 ± 37	93	114
Enniatin B1	0.2	83 ± 11	79	104
Enniatin B2	0.8	80 ± 2	88	91
Enniatin B3	0.1	90 ± 1	97	92
Enniatin B4	0.1	83 ± 2	99	84
Enniatin K1	0.3	89 ± 18	87	102
Equisetin	15	193 ± 7	231	84
Fumonisin B_1_	200	37 ± 1	107	34
Fumonisin B_2_	220	34 ± 3	110	31
HC toxin ^d^				
HT-2 toxin	55	96 ± 7	96	100
Kojic acid	230	28 ± 1	32	90
Macrosporin	3.1	172 ± 17	171	101
Meleagrin	19	80 ± 2	67	120
3-*O*-Methylsterigmatocystin	7.4	91 ± 3	98	93
Moniliformin	17	16 ± 1	19	80
Mycophenolic acid	26	92 ± 4	95	97
Neoxaline^d^				
Nidulin	1.6	62 ± 2	98	63
3-Nitropropionic acid	19	39 ± 1	44	87
Nivalenol	95	6 ± 2	3	189
Nornidulin	1.4	105 ± 3	141	74
Ochratoxin A	18	79 ± 3	94	84
Ochratoxin B	10	75 ± 1	86	87
Penigequinolone A	1.3	132 ± 4	162	82
Pestalotin	6.9	93 ± 3	93	99
Physcion	76	150 ± 6	246	61
Pseurotin A	28	156 ± 13	185	84
Roquefortine C	5.6	111 ± 1	81	137
Secalonic acid D	48	73 ± 5	110	67
Sterigmatocystin	2.7	93 ± 5	94	99
T-2 toxin	5.5	95 ± 5	97	98
Tentoxin	1.1	101 ± 2	108	94
Viridicatin	8.0	94 ± 0.2	97	97
Zearalenone	6.1	104 ± 2	117	89
Zearalenone-14-sulphate	5.6	67 ± 5	97	69

*LLOQ* lower limit of quantification

^a^Apparent recovery and standard deviation thereof (triplicate analysis on medium level)

^b^Mean value for the signal suppression or enhancement calculated from up to seven concentration levels without replicates

^c^Calculated extraction recovery (*R*_A_/SSE × 100)

^d^Due to interferences, it was not possible to evaluate HC toxin and neoxaline in pistachios

In our case, the obtained standard deviation of *R*_A_ was below 10 % for the vast majority of the analyte–matrix combinations. In only 6 % of the cases were values above 15 % determined, e.g. for enniatin A (13–23 %) and macrosporin (6–25 %). Significant deviations from 100 % apparent recovery were either caused by severe matrix effects or insufficient extraction. Fifty-seven per cent of the analyte–matrix combinations showed SSE values between 80 and 120 %. Suppression of the analyte signal to more than the half compared to the neat standard was observed for early-eluting analytes 3-nitropropionic acid, moniliformin, kojic acid, as well as for the B-trichothecenes (nivalenol, DON and 3-acetyldeoxynivalenol) in all four matrices. Significant signal enhancement of more than 120 % was observed for up to 17 analytes (almonds). The highest values were observed for physcion (246–295 %) and equisetin (231–285 %). Interestingly, for β-zearalenol-14-glucoside, a signal suppression to 54 and 79 % for peanuts and almonds, respectively, were determined, whereas no matrix effect was monitored for pistachios (97 %) and a significant signal enhancement of 127 % was observed in hazelnuts. This emphasises the importance of proper validation and indicates that samples belonging to the same food category can still show quite different matrix effects. The used solvent (acetonitrile/water/acetic acid (79:20:1, *v*/*v*/*v*) was well suited for the extraction of almost all analysed mycotoxins from the four different commodities. Ninety-four per cent of the analyte–matrix combinations showed extraction recoveries higher than 50 %. Lower *R*_E_ values were for example observed for fumonisins (29 and 42 %), which were even lower than reported before for wheat (43–53 %) and maize (57–67 %) using the same solvent mixture for extraction [4]. In cases of severe matrix suppression, e.g. SSE of 8 % for DON in pistachios, also unreliable *R*_E_ values were calculated (e.g. 225 % for DON in pistachios).

The linear range covered two orders of magnitude in most cases. For atpenin A5, even a linear range of three orders of magnitude was observed in all four matrices. The LLOQs varied between sub-microgram per kilogram levels, e.g. for apicidin, beauvericin and enniatins, and up to 500 μg kg^−1^ for HC toxin in hazelnuts. It should be pointed out here that in some cases (e.g. altertoxin I, atpenin A5 or neoxaline), even the lowest spiking level showed an S/N ratio above 10. All shown LLOQs will undoubtedly be higher compared to calculations based on S/N ratios equalling 10. We decided on purpose for this very conservative calculation of the LLOQs to minimize the influence of day-to-day performance differences, which are often encountered in mass spectrometry due to staining of the ion path. Still, the obtained recoveries and LLOQs were in a similar range as those reported before for peanuts [28], with the exception of DON due to signal suppression in our case. However, the gained standard deviations were lower in our proposed method, which can be partly explained by the combination of three different blank materials by Warth and co-workers, whereas we used one blank material and spiked it in triplicate. In comparison to [5], both for peanuts and pistachios, our method proved to be more repeatable for almost all analytes. The limits of detection were significantly lower for aflatoxins, OTA and fumonisins in the method proposed by Spanjer et al. [5], whilst they were lower for, e.g. zearalenone and T-2 toxin in our method. Compared to our method, lesser matrix effects were obtained by Ediage et al. [27] after extensive cleanup for 25 mycotoxins.

The gained LLOQs for aflatoxins are higher than the maximum levels set in Commission Regulation (EC) No. 1881/2006 and its amendments [3], rendering this method unsuited for regulatory purposes. Still, the presented method can be an appropriate supplement to already existing single-analyte or analyte-group detection methods which usually have been developed specifically for the respective target analytes in certain matrices. Alternatives to gain the needed sensitivity are cleanup and enrichment of aflatoxins (e.g. by immunoaffinity columns), a method parameter set which is optimised on those compounds, or the use of a more sensitive mass spectrometer [33]. Finally, albeit the method was validated for nuts, it is also applicable for the screening of mycotoxins in several other food and feed.

### Naturally contaminated nut samples

**Fig. 1:**
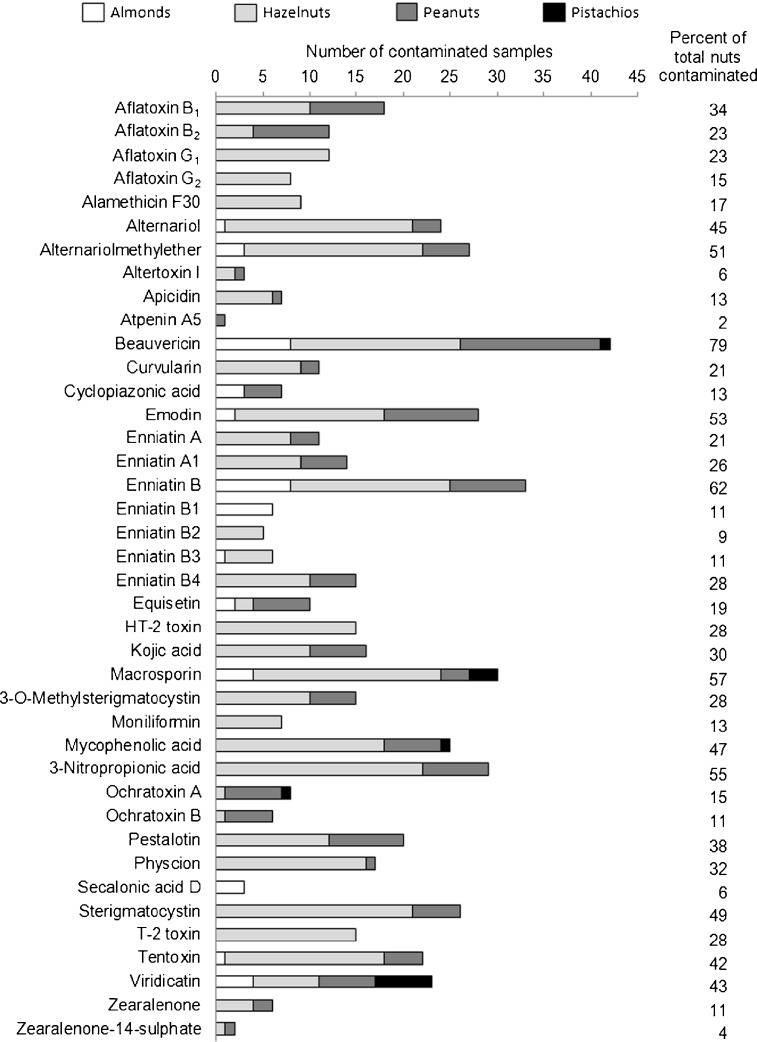
Overview of the detected mycotoxins in the analysed samples

Information about the occurrence of other mycotoxins than aflatoxins and OTA in nuts is very limited in the literature. We were able to show the applicability of the method through the analysis of 53 different nut (8 almonds, 22 hazelnuts, 15 peanuts and 8 pistachios) samples. Further microbial investigations about the source and time point of contamination are warranted and the scope of future experiments. Figure 1 provides an overview of the detected mycotoxins in the analysed samples and presents the percentage of contaminated nut samples compared to all analysed samples of the respective analyte. In Table 7, more detailed information including the average concentration of contaminated samples and the maximum observed level is given. In total, the presence of 40 analytes could be confirmed in different kinds of nuts. Most analytes were determined in hazelnuts (36), followed by peanuts (30), almonds (13) and pistachios (5). The reported mycotoxins are produced by a wide range of food spoilage and contaminating fungal species, including *Aspergillus* spp. (e.g. aflatoxins, sterigmatocystin); *Fusarium* spp. (e.g. ennitatins, equisetin); *Penicillium* spp. (e.g. mycophenolic acid, roquefortine C); and *Alternaria* spp. (e.g. alternariol, macrosporin). The most prevalent mycotoxin was beauvericin, which was identified in 42 samples, followed by enniatin B (33), macrosporin (30) and 3-nitropropionic acid (29). Beauvericin is a depsipeptide produced by various *Fusarium* spp.; concentrations up to 31 μg kg^−1^ in hazelnuts and 12 μg kg^−1^ in peanuts were observed. These values are in very good accordance to the occurrence of beauvericin in peanuts (0.1–24.0 μg kg^−1^, 73 % positive samples) reported before [28]. The contamination range was higher than reported by [26] in peanut cake (0.05–3.36 μg kg^−1^). Furthermore, several *Alternaria* mycotoxins were found in nuts, with alternariolmethylether (27 samples), alternariol (24 samples) and tentoxin (22 samples) being the most prevalent. Also tenuazonic acid was detected in 21 samples, with apparently high concentration, although this analyte was not part of the validated compounds. Emodin was found in 28 out of 53 samples, which is in agreement with the occurrence of this *Aspergillus* mycotoxin reported previously in inoculated peanuts [34]. In one hazelnut sample, 26 analytes were determined, and eight other hazelnut samples were contaminated with 20 or more mycotoxins. In peanuts, almonds and pistachios, up to 17, 13 and 5 analytes, respectively, were detected in one sample.

**Table 7:** Mycotoxin contamination of analysed nut samples

Analyte	Almonds (*n* = 8)		Hazelnuts (*n* = 22)		Peanuts (*n* = 15)		Pistachios (*n* = 8)	
	Avg.^a^ (μg kg^−1^)	Max.^b^ (μg kg^−1^)	Avg.^a^ (μg kg^−1^)	Max.^b^ (μg kg^−1^)	Avg.^a^ (μg kg^−1^)	Max.^b^ (μg kg^−1^)	Avg.^a^ (μg kg^−1^)	Max.^b^ (μg kg^−1^)
Aflatoxin B_1_	–	n.d.	7.4 (10)	15	68 (8)	230	–	n.d.
Aflatoxin B_2_	–	n.d.	5.5 (4)	<LLOQ	11 (8)	22	–	n.d.
Aflatoxin G_1_	–	n.d.	16 (12)	28	–	n.d.	–	n.d.
Aflatoxin G_2_	–	n.d.	5.5 (8)	<LLOQ	–	n.d.	–	n.d.
Alamethicin F30	–	n.d.	110 (9)	<LLOQ	–	n.d.	–	n.d.
Alternariol	1.5 (1)	<LLOQ	78 (20)	650	4.4 (3)	<LLOQ	–	n.d.
Alternariolmethylether	0.56 (3)	0.84	59 (19)	220	1.4 (5)	<LLOQ	–	n.d.
Altertoxin I	–	n.d.	7.0 (2)	<LLOQ	–	<LLOQ	–	n.d.
Apicidin	–	n.d.	3.4 (6)	14	–	<LLOQ	–	n.d.
Atpenin A5	–	n.d.	–	n.d.	–	<LLOQ	–	n.d.
Beauvericin	0.10 (8)	<LLOQ	2.4 (18)	31	1.6 (15)	12	0.30 (1)	<LLOQ
Curvularin	–	n.d.	19 (9)	42	19 (2)	24	–	n.d.
Cyclopiazonic acid	64 (3)	130	–	n.d.	140 (4)	310	–	n.d.
Emodin	1.7 (2)	<LLOQ	5.5 (16)	23	5.5 (10)	<LLOQ	–	n.d.
Enniatin A	–	n.d.	28 (8)	170	1.4 (3)	<LLOQ	–	n.d.
Enniatin A1	–	n.d.	140 (9)	1,100	0.70 (5)	<LLOQ	–	n.d.
Enniatin B	0.05 (8)	<LLOQ	37 (17)	540	0.23 (8)	0.45	–	n.d.
Enniatin B1	0.12 (6)	0.21	–	n.d.	–	n.d.	–	n.d.
Enniatin B2	–	n.d.	3.0 (5)	8.9	–	n.d.	–	n.d.
Enniatin B3	0.05 (1)	0.05	0.06 (5)	0.11	–	n.d.	–	n.d.
Enniatin B4	–	n.d.	22 (10)	190	0.1 (5)	<LLOQ	–	n.d.
Equisetin	8.5 (2)	<LLOQ	110 (2)	200	14 (6)	41	–	n.d.
HT-2 toxin	–	n.d.	39 (15)	130	–	n.d.	–	n.d.
Kojic acid	–	n.d.	1,100 (10)	1,400	8,900 (6)	40,000	–	n.d.
Macrosporin	2.1 (4)	4.4	280 (20)	2,200	9.4 (3)	26	4.6 (3)	11
3-*O*-Methyl-sterigmatocystin	–	n.d.	1.7 (10)	3.9	1.6 (5)	2.7	–	n.d.
Moniliformin	–	n.d.	5.8 (7)	9.2	–	n.d.	–	n.d.
Mycophenolic acid	–	n.d.	700 (18)	6,100	21 (6)	60	13 (1)	<LLOQ
3-Nitropropionic acid	–	n.d.	440 (22)	980	82 (7)	350	–	n.d.
Ochratoxin A	–	n.d.	220 (1)	220	67 (6)	260	9.0 (1)	<LLOQ
Ochratoxin B	–	n.d.	6.9 (1)	6.9	31 (5)	82	–	n.d.
Pestalotin	–	n.d.	3.1 (12)	<LLOQ	5.4 (8)	21	–	n.d.
Physcion	–	n.d.	700 (16)	3,300	–	<LLOQ	–	n.d.
Secalonic acid D	31 (3)	51	–	n.d.	–	n.d.	–	n.d.
Sterigmatocystin	–	n.d.	2.3 (21)	5.5	1.2 (5)	<LLOQ	–	n.d.
T-2 toxin	–	n.d.	32 (15)	40	–	n.d.	–	n.d.
Tentoxin	0.60 (1)	<LLOQ	5.4 (17)	21	4.7 (4)	11	–	n.d.
Viridicatin	3.8 (4)	<LLOQ	5.7 (7)	15	3.9 (6)	<LLOQ	4.0 (6)	<LLOQ
Zearalenone	–	n.d.	7.6 (4)	21	2.9 (2)	<LLOQ	–	n.d.
Zearalenone-14-sulphate	–	n.d.	3.9 (1)	<LLOQ	2.6 (1)	<LLOQ	–	n.d.

*n* total number of samples analysed in this category, *n*.*d*. not detected, <*LLOQ* below the lower limit of quantification

^a^Average of samples with detectable amounts of the specific analyte; in parentheses, the number of samples used for the calculation is given (in the case of detectable levels which are below the LLOQ, half of the LLOQ of the respective matrix was used for calculation)

^b^Highest determined concentration

Almond and especially pistachio samples were contaminated to a lesser extent than peanut and hazelnut samples. However, it has to be kept in mind that the sample size was also smaller for those matrices. In the case of pistachios, only five analytes could be determined and only one sample was contaminated above the LLOQ (11 μg kg^−1^ macrosporin). Concerning almonds, 15 analytes were determined, among others *Alternaria* toxins and enniatins. The highest contamination was observed for cyclopiazonic acid (up to 130 μg kg^−1^) and secalonic acid D (up to 51 μg kg^−1^).

Some hazelnut samples showed significant contamination, including aflatoxins (up to 15 μg kg^−1^ AFB_1_ and 28 μg kg^−1^ AFG_1_) and *Alternaria* mycotoxins (up to 650 μg kg^−1^ alternariol and 220 μg kg^−1^ alternariolmethylether). The determined AFB_1_ concentrations were above the maximum level set in the European Union (2.0/5.0/8.0/8.0 μg kg^−1^ AFB_1_ in peanuts, hazelnuts, almonds and pistachios, respectively) [3] for eight hazelnut and eight peanut samples. 3-Nitropropionic acid (up to 980 μg kg^−1^) was determined in all hazelnut samples. Sterigmatocystin (up to 5.5 μg kg^−1^), the most toxic AFB_1_ precursor, was found in 21 out of the 22 analysed hazelnut samples. We were also able to identify T-2 and HT-2 toxins in nut samples for the first time ever. Whereas the occurrence of these toxins was never confirmed in nuts, several *Fusarium* spp. including the T-2- and HT-2-producing *Fusarium oxysporum* were detected on almonds and pistachios before [35]. Average values of the contaminated 15 samples were 39 and 32 μg kg^−1^ for the HT-2 and T-2 toxins, respectively. In 17 hazelnut samples, enniatin B was detected reaching levels up to 540 μg kg^−1^. The highest concentration of all enniatins was about 1100 μg kg^−1^ in a single hazelnut sample, whilst in no other commodity were enniatins found above 0.5 μg kg^−1^. With regard to mycophenolic acid, a potent immunosuppressive compound, one sample was contaminated with 6,100 μg kg^−1^. In addition, macrosporin was found up to 2,200 μg kg^−1^.

In peanuts, 32 analytes were detected. The highest contamination was observed for a sample containing 40 mg kg^−1^ kojic acid; the most prevalent was beauvericin, which was detected in all samples with an average concentration of 1.6 μg kg^−1^ and a maximum of 12 μg kg^−1^. As already pointed out in [28, 36], we could confirm the presence of cyclopiazonic acid, a mycotoxin produced, e.g. by *Aspergillus flavus*, in four peanut samples up to a concentration of 310 μg kg^−1^. This is a lower contamination rate as reported before [28, 36]. Also, for 3-nitropropionic acid, previously determined concentrations [28] have been verified.

## Conclusion

In conclusion, an UHPLC-MS/MS-based method for the determination of 191 mycotoxins and other fungal metabolites has been developed. Compared to other LC-MS/MS methods, UHPLC allowed better separation of the analytes from the matrix. Whilst most methods for the determination of mycotoxins focus on cereals or cereal-based foods, we developed a method for almonds, hazelnuts, peanuts and pistachios. An in-house validation for 65 analytes was performed, allowing the quantification of those analytes in the four commodities. For the other 126 analytes, the method still can provide semiquantitative information about the degree of contamination, and additional analytes might be validated afterwards, if needed. The overall repeatability of the proposed method is superior to currently published methods.

The method is based on a fast and easy sample preparation, including a single extraction step and subsequent injection of the diluted raw extract into the UHPLC-MS/MS system without any sample cleanup. Two chromatographic runs for each sample allow a throughput of about 25 samples per day (including standards). As with all multi-target methods, the major bottleneck regarding sample throughput is data evaluation, which is quite laborious and time-consuming. Various software tools, in particular “compound at a glance” or flagging options, can greatly assist in this process, though.

Finally, the method has been applied to the analysis of 53 different nut samples. In total, 40 different analytes were detected, showing the importance of multi-mycotoxin methods. It seems that besides aflatoxins, the only mycotoxins regulated in nuts in the European Union, other toxins might also be relevant. The obtained mycotoxin pattern shows that a variety of fungal species, including *Aspergillus* spp., *Penicillium* spp., *Fusarium* spp. and *Alternaria* spp., might grow on nuts and are capable of producing a variety of toxins. The most prominent mycotoxins found in more than 50 % of the samples were beauvericin, ennitatin B, macrosporin, 3-nitropropionic acid, emodin and alternariolmethylether. Additionally, we could, for the first time, confirm the presence of HT-2 and T-2 toxin in hazelnuts. For many of the detected mycotoxins, possible toxic effects on humans are still not fully evaluated. Even more, possible additive or synergistic effects of co-occurring toxins are largely unknown. The major benefit of the developed method is its usage in the (semi-)quantitative screening for a large number of mycotoxins and other fungal metabolites in nuts and food in general.
